# Bilateral Orbital Metastases Masquerading as Ocular Myasthenia Gravis: A Case Report and Review of the Literature

**DOI:** 10.7759/cureus.9105

**Published:** 2020-07-09

**Authors:** Dario A Marotta, Maxwell J Jabaay, Adena Zadourian, Hassan Kesserwani

**Affiliations:** 1 Department of Research, Alabama College of Osteopathic Medicine, Dothan, USA; 2 Department of Neurology, Division of Neuropsychology, University of Alabama, Birmingham, USA; 3 Neurology, Flowers Medical Group, Dothan, USA

**Keywords:** ocular myasthenia gravis, orbital metastases, cancer

## Abstract

Ocular myasthenia gravis and orbital metastases have overlapping symptoms but divergent diagnostic and treatment strategies. Here, we present a 58-year-old female, with a 20-year history of advanced metastatic breast cancer, who presented to the neurology clinic with fatigue, muscle weakness, bilateral ptosis, and diplopia that worsened throughout the day. While the initial presentation was consistent with ocular myasthenia gravis, a subsequent evaluation revealed bilateral metastatic lesions of breast origin within the orbits. This case highlights the variable nature of metastatic disease and underscores the importance of a comprehensive neoplastic workup in patients with new-onset symptomatology and a prior history of advanced cancer despite purported remission status.

## Introduction

In the United States, breast cancer remains the second most fatal cancer for women, claiming the lives of 40,000 of the 300,000 women diagnosed each year [1]. Six percent to 30% of breast cancers will metastasize, most commonly to the bone, lung, liver, and brain [2-3]. A change from baseline in a patient with a prior history of cancer may suggest re-occurrence or metastatic spread. However, such presentations can be variable and can mimic many other disease processes. For instance, brain metastases can manifest as mood aberrations and cognitive decline, thereby mimicking anxiety, depression, and other mood-related disorders [4-5]. Metastatic spread to other locations, such as the orbits, can cause diplopia, ptosis, and even pain; which may resemble multiple sclerosis, myasthenia gravis, and even cerebrovascular incidents [6-7]. In this case, we report a 58-year-old female with a longstanding history of metastatic breast cancer. The patient’s initial symptoms are consistent with ocular myasthenia gravis, but a thorough evaluation revealed a more ominous cause. This case showcases the variable manifestations of metastatic disease and underscores the importance of a comprehensive neoplastic workup in the setting of advanced cancer.

## Case presentation

A 58-year-old female presented to the neurology clinic after experiencing six months of progressive fatigue, diffuse muscle weakness, dysphagia, bilateral eye pain, vertical diplopia, and ptosis. The patient’s symptoms reportedly worsened in the afternoons. Her medical history included a 20-year history of metastatic breast cancer, gastric neuroendocrine carcinoma, insulin-dependent diabetes, cirrhosis of the liver, and asthma. The patient had undergone a double mastectomy before suffering a malignant pleural effusion and subsequent metastatic spread to the sternum in the last five years. Testing within the past two years suggested the absence of active neoplastic disease. The patient’s medication included anastrozole 1 mg daily, gabapentin 800 mg three times daily, hydroxychloroquine 200 mg twice daily, ophthalmic ketorolac 0.5%, lanreotide, insulin, subcutaneous denosumab 120 mg/1.7 mL every four weeks, and oxycodone and albuterol as needed.

Physical examination revealed that the patient was able to ambulate independently and without significant disturbances in gait or balance. Bilateral ptosis was observed, more prominent on the right. Hypertropia of the right eye was noted, but it fluctuated during exam. Repetitive blinking worsened the hypertropia. There was no evidence of abnormal pupillary size or reaction to light, horizontal or vertical nystagmus, or extraocular muscle deficits. Funduscopic examination by an ophthalmologist did not reveal papilledema. Visual acuity was 20/20 in both eyes. Using the Medical Research Council grading scale, muscle strength testing showed no weakness (5/5) throughout. Likewise, deep tendon reflexes were 2+ and symmetrical bilaterally. The patient was screened for ocular myasthenia gravis and started on pyridostigmine at an initial dose of 60 mg three times daily. However, the patient did not respond to empiric therapy and her symptoms persisted.

**Figure 1:**
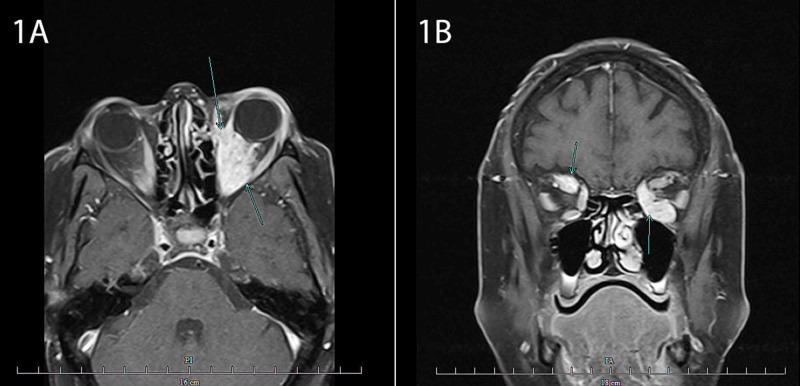
Orbital Magnetic Resonance Imaging 1A: Axial MRI of the orbits with contrast revealing a left mass lesion (blue arrows). 1B: Coronal MRI of the orbits with contrast revealing a left soft tissue mass engulfing the medial and inferior extraocular muscles and a right soft tissue mass involving the superior rectus muscle (blue arrows). MRI: Magnetic Resonance Imaging

Given the lack of response to pyridostigmine and due to a history of metastatic breast cancer, magnetic resonance imaging (MRI) of the brain and orbits with and without contrast was obtained (Figure 1). This revealed a left orbit mass measuring 1.4 x 2.4 cm that engulfed the medial and inferior extraocular muscles and a right orbit mass measuring 1.4 x 1.4 cm that was intertwined with the superior rectus muscle. The optic globes, optic nerves, optic chiasm, and remaining extraocular muscles were intact within both orbits. Serology was negative for anti-acetylcholine receptor binding, blocking, and striational antibodies.

**Figure 2:**
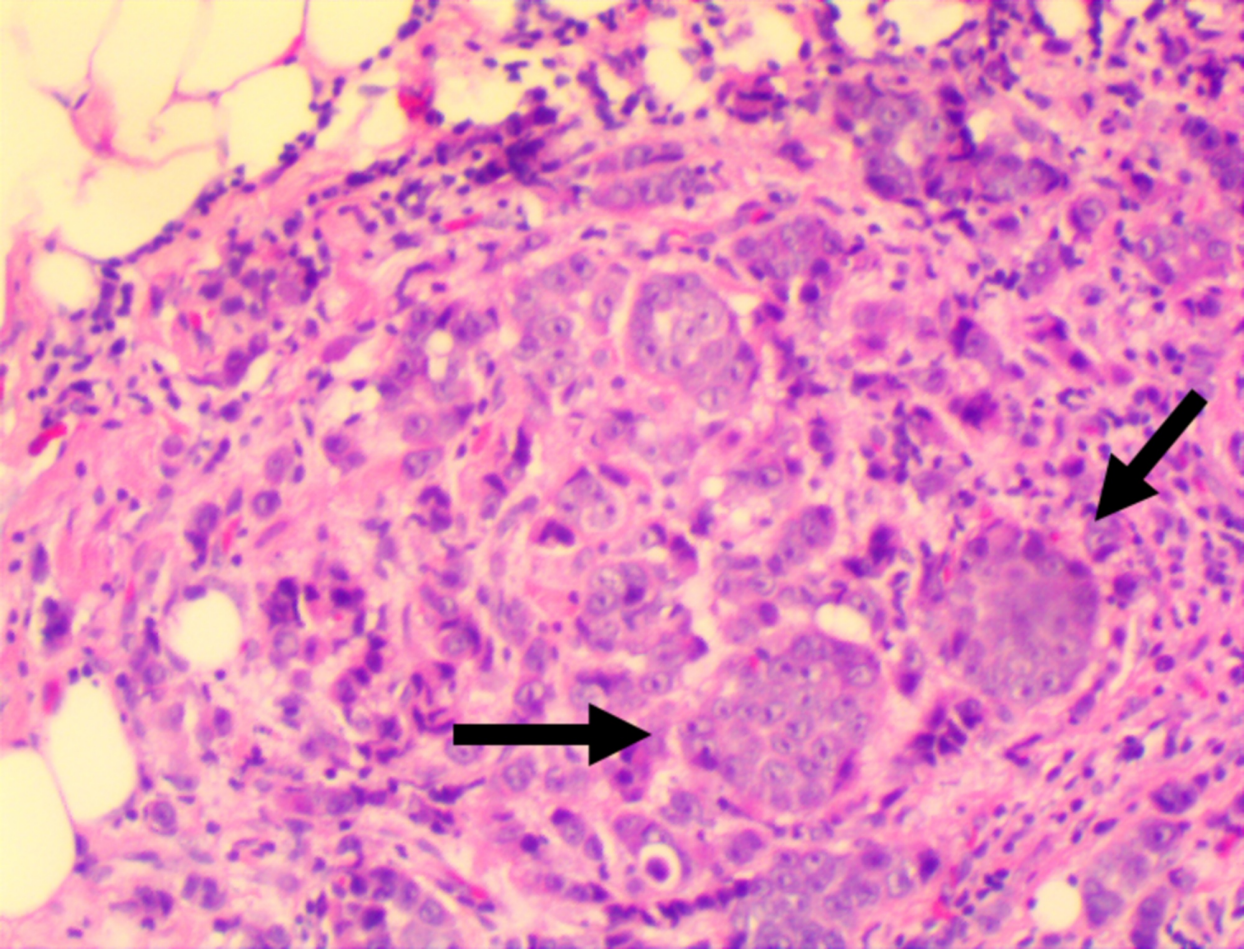
Hematoxylin and Eosin Staining of Left Orbital Lesion Biopsy Specimen Hematoxylin and eosin staining of the mass lesion biopsy from the patient's left orbit revealed fibroadipose tissue with several foci of malignant neoplasm characterized by cohesive intermediate to large cells arranged in nests (black arrows) with no definite gland formation.

The patient was referred to ophthalmology for an anterior orbitotomy and biopsy of the left orbital lesion. Hematoxylin and eosin staining of the mass lesion revealed fibroadipose tissue with several foci of malignant neoplasm characterized by cohesive intermediate to large cells that are arranged in nests with no definite gland formation (Figure 2). Subsequent staining of the mass lesion was positive for breast markers GATA-3, estrogen receptors, and mammaglobin. Together, these findings are consistent with metastatic carcinoma originating from the breast. The patient was initiated on a chemoradiation therapy regimen designed to preserve eye function. The regimen included intravenous infusions of ado-trastuzumab, pertuzumab, trastuzumab, and docetaxel. The patient also received radiation at 4500 cGy in 25 fractions over five weeks with intensity-modulated radiation therapy (IMRT) technology directed at the extraocular muscles. Special care was taken to avoid the lacrimal glands and anterior structures of the eyes. After three months of treatment, the patient noted a near-complete resolution of her diplopia. The patient remains under observation and palliative care. A repeat MRI of the orbits was scheduled at follow-up.

## Discussion

Myasthenia gravis is a relatively common autoimmune disorder of the neuromuscular junction with multiple distinct subtypes. The disease can be purely ocular or generalized. There are several serotypes with different semiology and therapeutic response to different immune-modulatory therapy. Population-based epidemiological studies comparing generalized and purely ocular myasthenia gravis are sparse, but a recent study revealed an estimated incidence of 2.2 and 1.1 per 100,000 persons per year, respectively [8]. The age at diagnosis is variable among subtypes. For ocular myasthenia gravis, the average age of onset has been estimated at 59 years [8]. Disease manifestations are commonly sequestered to the extraocular muscles but may transition to a systemic or generalized subtype over time. Fifty-two percent of patients with purely ocular myasthenia gravis experience extraocular weakness, ptosis, and diplopia with increasing fatigability throughout the day [9]. Patients with these hallmark characteristics and clinical presentations should prompt a high index of suspicion for ocular myasthenia gravis. However, as demonstrated with this case report, the differential diagnosis should be expanded to incorporate a comprehensive neoplastic workup in the presence of a history of other co-morbid illnesses such as malignancy.

One in eight women in the United States will be diagnosed with invasive breast cancer; 10% of these will be estrogen receptor (ER) and human epidermal growth factor receptor 2 (HER2) positive [2]. These genetic profiles confer somewhat of a survival advantage, which has continued to improve over time. ER-positive metastatic breast cancer (MBC) patients saw median survival increase from 32 to 57 months between 1990 and 2010. In that same time period, median survival for ER-negative MBC patients increased from 14 to 33 months [10]. Nevertheless, metastatic spread remains a considerable risk to overall survival. Metastatic spread to the orbits is rare, occurring in 2% to 3% of patients [11]. In patients with orbital metastases, breast cancer was the most common tumor origin [6]. Patients with orbital metastases tend to carry poorer prognoses, and a reduction in average survival is estimated at between 10 and 31 months [11]. Therefore, the development of acute symptoms in patients with complex cancer histories should signal the potential for metastatic spread. Rapid identification of these lesions may help reduce morbidity and mortality while improving the remaining quality of life through palliative care.

Differentiating ocular myasthenia gravis from bilateral orbital metastases on a purely clinical basis can be challenging since many symptoms overlap. Similar to patients with ocular myasthenia gravis, patients with bilateral metastases to the orbit may present with diplopia, proptosis, and ocular motility deficits [6]. The presence of pain, as described by the patient in this case, may suggest metastatic involvement, although this finding is subjective and variable between patients [7]. Nevertheless, in patients with a history of advanced cancer, the use of computed tomography (CT) or magnetic resonance imaging (MRI) can be used to reveal the presence of mass lesions. However, even in the absence of findings on CT or MRI, alternative techniques may be available for detection such as optical coherence tomography. In contrast, confirmation of ocular myasthenia typically involves repetitive nerve stimulation testing or autoantibody serology for anti-acetylcholine receptor antibodies, anti-muscle-specific tyrosine kinase (anti-MuSK) antibodies, anti-titin antibodies, and anti-low density lipoprotein receptor-related protein 4 antibodies [12]. These tests may be negative even in the presence of disease [13]. This is especially true for patients with ocular myasthenia gravis; only 33% of patients show a decremental response to repetitive nerve stimulation, and only 33% to 77% demonstrate the presence of anti-acetylcholine receptor antibodies [14-16].

Treatment strategies between ocular myasthenia gravis and orbital metastases are as divergent as the diagnostic strategies. For patients who display the characteristic symptoms of ocular myasthenia gravis, pyridostigmine remains the drug of choice. In addition to effectiveness and rapid onset, response to treatment can function as an alternative diagnostic tool [17]. Corticosteroids have also been shown to produce favorable responses in a majority of patients with ocular myasthenia gravis [18]. However, treatment onset for corticosteroids is comparatively longer and may present additional implications in patients with complex medical histories. In contrast to ocular myasthenia gravis, treatment for orbital metastases is generally palliative [19]. Extended durations of chemotherapy and anastrozole adjuvant therapy may improve survival outcomes to a degree. However, it is unclear how these treatments impact survival by the time orbital metastases are identified [20]. In these instances of advanced-stage cancer, prompt referrals to radiation oncology experts is critical to establish an evidence-based treatment and/or palliative care regimen.

## Conclusions

Ocular myasthenia gravis and bilateral orbital metastases may present with similar clinical characteristics. Disease identification and management is complicated by overlapping symptoms, divergent diagnostics, and differing treatment strategies. In this case, we report a 58-year-old female with bilateral orbital metastases presenting as ocular myasthenia gravis. This case illustrates the need for neoplastic considerations in patients with a prior history of advanced cancer, despite the presence of longstanding remission.
